# Reciprocal Interactions Between Periodontal Disease and Alzheimer’s Disease: Implications for Mutual Triggering, Exacerbation, and Treatment Interventions—A Comprehensive Review of the Literature

**DOI:** 10.3390/neurolint17060081

**Published:** 2025-05-24

**Authors:** Shatha Gharaibeh, Alameen Alsabbah, Ahmad Alloubani, Abeer Gharaibeh

**Affiliations:** 1 Division of Periodontics, Ministry of Health, Amman 11118, Jordan; shatha.gharaibeh@iinn.com; 2 Insight Research Institute, Flint, MI 48507, USA; alameen.alsabbah@iinn.com (A.A.); ahmad.alloubani@iinn.com (A.A.); 3 Center for Cognition and Neuroethics, University of Michigan-Flint, Flint, MI 48502, USA; 4 Insight Institute of Neurosurgery & Neuroscience, Flint, MI 48507, USA; 5 Insight Hospital and Medical Center, Chicago, IL 60616, USA

**Keywords:** Alzheimer’s, periodontitis, periodontal disease, cognitive, biomarkers, neuroinflammation, neurodegenerative

## Abstract

Periodontal health is connected to many systemic diseases, such as cardiovascular, diabetes mellitus, and neurodegenerative diseases. The oral–brain axis has gained increasing interest in the pathogenesis of diseases. Emerging studies have highlighted the potential role of periodontal disease in the development and progression of Alzheimer’s disease. However, Alzheimer’s disease also affects periodontal disease and oral health. In this review, we address the correlation between the two diseases and the mechanisms by which one contributes to the other. Exploring the correlation between Alzheimer’s disease and periodontal disease will assist in better understanding the pathophysiology of diseases and pave the way for the development of therapeutic and preventive strategies.

## 1. Introduction

Alzheimer’s disease (AD) is a complex and multifactorial neurodegenerative disorder [1,2]. A hallmark of AD is a gradual decline in cognitive functions, which are lost in the severe and final stages of AD [3]. AD is trending towards being one of the most expensive and lethal diseases of the century [3]. Currently, it is the most common cause of dementia, accounting for 60–70% of all cases worldwide [4,5]. Dementia is a consequential cause of disability and dependency in older adults, and it is the seventh leading cause of death worldwide [4,5,6,7]. In 2019, the global economic impact of dementia hit USD 1.3 trillion, and this is expected to reach USD 2.8 trillion by 2030 [4,5].

The diagnosis of AD is challenging and primarily relies on clinical evaluations, which consist of a detailed patient history, a full clinical assessment, cognitive function tests, and imaging studies [1,2]. At present, there are no clinically available tests that can be used to diagnose AD before the appearance of symptoms, by which point brain damage has already occurred [1,3]. However, multiple risk factors are known to influence AD development, including genetics and environmental risk factors, such as age, chronic vascular diseases, and obesity [2]. Twin studies have estimated the heritability of AD to be between 60 and 70% [3]. Furthermore, genome-wide studies and transcriptomic data have identified several genes associated with the risk of developing AD, thus shedding light on novel pathological pathways involved in its development [3].

Classically, AD pathogenesis is associated with abnormal protein aggregations of amyloid β (Aβ) and tau, called amyloid plaque and neurofibrillary tangles [8]. These are considered the core pathological markers of AD and are believed to be the main drivers of neuronal death in the disease [8]. Earlier research initially focused on the role of these pathological hallmarks in mechanism studies and drug development. However, thorough investigations of AD and its mechanisms have identified new players and molecular targets in the disease, including aberration in mitochondrial function, vascular integrity, synaptic and neurotransmitter signaling, and neuroinflammation [1].

AD research extends beyond understanding its mechanisms to identifying novel biomarkers, molecular targets, prevention strategies, and additional risk-modifying factors [3]. While certain risk factors are widely reported, others—such as periodontal disease—have received less attention [1]. Emerging studies have highlighted the potential role of periodontal disease in the development and progression of AD [9]. Periodontitis, which is considered an advanced stage of periodontal disease, is an inflammatory disease of the periodontium [10]. It is initiated by bacterial pathogens when the balance between the host and microbial factors is disrupted, leading to dysbiosis and destructive inflammation [10]. Environmental, physical, social, and host stresses modify periodontitis through a multitude of pathways [10]. Periodontal disease affects about 20–50% of the global population [11]. In the United States, around 42.2% of adults aged 30 years have periodontal disease [12]. The high prevalence of periodontal disease makes it a public health concern [11].

Historically, the dentist and oral microbiologist Willoughby D. Miller has long asserted that “the human mouth, as a gathering-place and incubator of diverse pathogenic germs, performs a significant role in the production of varied disorders of the body, and that if many diseases whose origin is enveloped in mystery could be traced to their source, they would be found to have originated in the oral cavity” [13]. Systemic conditions can lead to the initiation and progression of periodontal disease [9,11,14,15]. However, the potential effects of periodontal disease on systemic health are being documented more than ever before [15]. Periodontal disease is significantly associated with many systemic diseases, including coronary heart disease, diabetes, perinatal conditions, pulmonary disease, neurodegenerative disorders, and cancer [15,16,17,18,19,20]. Host susceptibility to periodontal pathogens is important in understanding the differences in the progression of periodontal disease and its effect on systemic health [21,22].

Studies have suggested a reciprocal relationship between AD and periodontal disease [14,20,23,24]. Therefore, understanding the potential link between periodontal disease and AD is crucial for the development of new therapeutic and preventive strategies [25,26]. To further explain this, periodontal disease presents with a polymicrobial etiology and amplifies low-grade systemic inflammation [14,19,25,27]. This systemic inflammatory process is facilitated by the entry of periodontal bacteria and their components into the systemic circulation [9,28,29]. The systemic inflammation caused by periodontal disease has been implicated in exacerbating cognitive decline [20,30].

In this paper, we provide a comprehensive review of the correlation between periodontal disease and AD, the biomarkers for AD with periodontal disease, and the existing correlated therapeutic modalities between both diseases. The goal of this review is to increase the awareness of physicians and the scientific community about this connection and facilitate preventive and treatment studies.

## 2. Methods

To identify studies relevant to the biological and clinical bidirectional relationship between periodontal disease and AD, a literature search of electronic databases, including PubMed/MEDLINE, Scopus, and Google Scholar, was performed. We used keywords such as “Alzheimer”, “Periodontitis”, “cognition”, and “biomarker”, and their variations, with no restriction on the year of the publication. We included studies that were available in the English language and published in peer-reviewed journals. Specifically, we included data and conclusions focusing on the relationship between periodontal disease and AD and the bidirectional impact of either at the biological or clinical level. The literature review focused on studies that examined biomarkers for use in the screening of AD in patients with periodontal disease and that thoroughly discussed such biomarkers. Animal studies with results on biomarkers were also included. Editorials, conference abstracts, or case reports were excluded. The final included studies were chosen after a full-text screening of highly related articles that were retrieved after the initial assessment based on titles and abstracts. Due to the variety of study outcomes, populations, and designs, a narrative synthesis approach was used to extract and paraphrase the relevant information.

## 3. Discussion

### 3.1. Pathogenesis of AD

**Figure 1:**
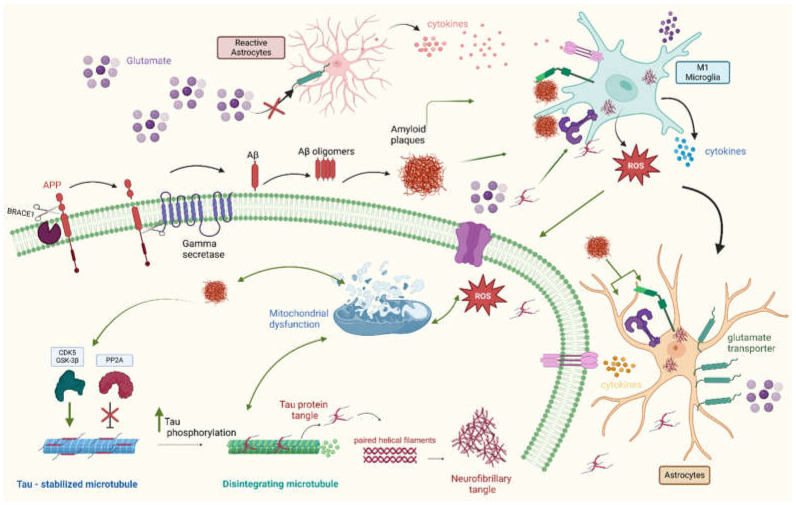
The pathogenesis of Alzheimer’s disease (AD). A hallmark of AD is the accumulation of amyloid-beta plaques. Initially, beta-site APP cleaving enzyme 1 (BACE1) cleaves amyloid precursor protein (APP) into a soluble APPβ fragment and a membrane-bound C-terminal fragment (CTFβ). Then, gamma-secretase cleaves CTFβ within the membrane, releasing Aβ peptides, which form soluble Aβ peptide oligomers that later convert into insoluble plaques. Another hallmark is the accumulation of microtubule-stabilizing tau protein and its aggregation to form neurofibrillary tangles. In AD, the increased activity of tau phosphorylases such as GSK-3B and CDK5 and the inhibition of PP2A phosphatase both lead to tau hyperphosphorylation. Tau hyperphosphorylation promotes the formation of tau tangles and the disintegration of microtubules. Tau tangles then form paired helical filaments and neurofibrillary tangles, which are toxic to neurons. Amyloid plaques also promote tau phosphorylation by activating several phosphorylases. Both plaques and neurofibrillary tangles further contribute to AD pathogenesis by promoting neuroinflammation and other cellular changes, such as mitochondrial dysfunction. Neuroinflammation plays an active role in AD pathogenesis. A microglial polarization imbalance towards the M1 phenotype releases more pro-inflammatory cytokines and ROS, which further promote neuroinflammation and induce neurotoxicity. Microglial cytokines activate astrocytes, which, in turn, become reactive and release inflammatory mediators that activate microglia. Reactive astrocytes express lower numbers of glutamate transporters, resulting in the accumulation of glutamate, which induces neurotoxicity. Both plaques and tau neurofibrillary tangles promote inflammation by binding to inflammatory receptors such as receptors for advanced glycation end products (RAGEs) and Toll-like receptors (TLRs) on microglia and astrocytes, which, in turn, respond by releasing pro-inflammatory cytokines and reactive oxygen species (ROS). Another feature is mitochondrial dysfunction, which is induced by amyloid plaques and neurofibrillary tangles. Mitochondrial dysfunction reduces synaptic energy, promotes neuronal death, and further promotes ROS and pathological protein aggregation. Created in BioRender. Gharaibeh, A. (2025): https://BioRender.com/j8v9kpq (accessed on 21 May 2025).

Broadly, AD is divided into late-onset sporadic and early-onset familial forms [31]. The familial form is mainly caused by mutations in genes that regulate amyloid and tau proteins, leading to their pathological aggregations [31]. These aggregates are considered the core pathological features and constitute the fundamental hypothesis of AD development [1,31,32]. However, the sporadic form is more complex, involving the interplay of different cellular and extracellular components in the disease pathogenesis [1,31]. Deep investigations have revealed new insights into the mechanisms of AD pathogenesis that are explained in the multiple sections below and summarized in Figure 1.

#### 3.1.1. Amyloid and Tau Proteins

A central feature of the pathogenesis of AD is the accumulation of Aβ plaques in the brain [8]. Aβ peptides are produced by the amyloid precursor protein (APP) through sequential proteolytic cleavages by beta-site APP cleaving enzyme 1 (BACE1) and gamma-secretase [8,31]. Initially, BACE1 cleaves APP into a soluble APPβ fragment and a membrane-bound C-terminal fragment (CTFβ) [14,31,32,33]. Then, gamma-secretase cleaves the CTFβ within the membrane, releasing Aβ peptides of varying lengths, predominantly Aβ40 and Aβ42 [14,32,33]. However, Aβ42 is considered more pathogenic due to its aggregation [34,35,36].

The aggregation process of Aβ peptides is influenced by multiple factors, including their post-translational modifications, concentrations, and interactions with other peptides and ions [35,36]. Initially, Aβ peptides aggregate as soluble oligomers; these are then converted into insoluble fibrils, which accumulate as plaques extracellularly [32,34,36,37]. Aβ oligomers are considered to be the most toxic species, as their toxic effect starts even before plaque formation [1,34,37].

Another key protein in AD is tau, which is a microtubule-associated protein primarily expressed in neurons [38]. It promotes the assembly and stabilization of microtubules, which are essential for axonal transport and neuronal structure [38]. Tau is regulated by phosphorylation and normally undergoes reversible phosphorylation, which controls its binding to microtubules [39]. In AD, tau becomes abnormally hyperphosphorylated, leading to its detachment from microtubules and subsequent aggregation into paired helical filaments (PHFs) and neurofibrillary tangles (NFTs) [40]. Remarkably, phosphorylated tau, such as p-tau217, is gaining significant interest for being a highly accurate and sensitive plasma biomarker for AD, outperforming other imaging and available tests [41,42]. The hyperphosphorylation of tau is thought to be driven by the dysregulation of kinases, such as glycogen synthase kinase-3β (GSK-3β) and cyclin-dependent kinase 5 (CDK5), and phosphatases, such as protein phosphatase 2A (PP2A) [43].

NFTs are intracellular aggregates of hyperphosphorylated tau proteins after misfolding into soluble oligomers, which become insoluble fibers within the neuronal cytoplasm [44,45,46]. NFTs are predominantly found in the hippocampus, entorhinal cortex, and other brain regions involved in memory and cognition [47]. An accumulation of Aβ is believed to precede and promote tau phosphorylation and aggregation, enhancing the formation of NFTs [48]. Therefore, the combined presence of Aβ plaques and NFTs accelerates the disruption of neuronal function and neurodegeneration [49].

#### 3.1.2. Neuroinflammation in AD

Neuroinflammation is a key component of AD pathogenesis [50]. The process involves the activation of brain immune cells, including astrocytes and microglia, and the release of various inflammatory mediators [50]. Pro-inflammatory cytokines and reactive oxygen species (ROS) have pleiotropic effects in AD and are involved in different mechanisms of its pathogenesis. These include direct damage to neurons; (1) disruption of blood–brain barrier integrity; (2) increase in Aβ production and aggregation; (3) tau phosphorylation; (4) and activation of resident immune cells, such as microglia and astrocytes [50].

In AD, Aβ deposits activate microglia for phagocytosis via two important receptors: Toll-like receptors (TLRs) and receptors for advanced glycation end products (RAGEs) [51,52]. In response, microglia produce pro-inflammatory cytokines, such as IL-1β, IL-6, and tumor necrosis factor-alpha (TNF-α), as well as ROS [51,52]. The ability of microglia to perform phagocytosis is also diminished in AD, and this leads to plaque accumulation and the exacerbation of neuroinflammation [53]. Additionally, microglia are characterized by their ability to polarize into different phenotypes: a pro-inflammatory microglia (M1) state and an anti-inflammatory microglia (M2) state. In AD, there is an imbalance favoring the M1 state, which perpetuates inflammation and neurotoxicity [54].

Neurons release hyperphosphorylated tau proteins in a soluble form or as aggregates, which are taken up by microglia primarily through Fc and pattern recognition receptors (PRRs) [55]. The internalization of tau triggers microglia to produce pro-inflammatory cytokines and ROS [55,56]. Moreover, activated microglia can spread tau pathology throughout the brain by transferring tau aggregates to surrounding neurons [55,56]. This propagation feature is similar to that of prion disease [57]. The scientific community has agreed on the use of the term “prion-like disease” to describe AD and other neurodegenerative diseases with this feature [57].

Abundant microglial cells in the brain activate astrocytes. Upon activation, astrocytes undergo astrogliosis, a process that includes hypertrophy and proliferation around plaques and tangles [50,58]. This is accompanied by the release of inflammatory mediators, contributing to neuroinflammation [50,58]. Reactive astrocytes downregulate glutamate transporters and increase extracellular glutamate levels, causing excitotoxicity and neuronal damage [59]. Astrocytes can clear Aβ through endocytosis and degradation, but chronic Aβ exposure can impair this function. This leads to further accumulation of Aβ and sustained inflammation [60]. Astrocytes respond to extracellular tau similarly via astrogliosis and the release of inflammatory mediators after activation via PRRs [61]. Astrocytes can internalize tau, leading to the formation of tau aggregates within them [62]. Intracellular tau accumulation can disrupt astrocytic functions and further exacerbate neuroinflammation. Astrocytes lose their ability to support neuronal functions, including neurotransmitter regulation, blood–brain barrier (BBB) maintenance, and ion homeostasis. This contributes to neuronal dysfunction and death [63].

As a part of innate immunity, inflammasomes are multi-protein complexes that play a role in inflammatory responses [64]. The Node-Like Receptor Protein 3 (NLRP3) inflammasome has specifically been implicated in AD. In microglia and astrocytes, the NLRP3 inflammasome is activated in response to accumulated Aβ and hyperphosphorylated tau protein, leading to the activation of cysteine-dependent aspartate-directed protease caspase-1 [64]. Pro-inflammatory cytokines IL-1β and IL-18 are released and further contribute to the inflammatory process [64]. Activated astrocytes can influence microglial activity by releasing signaling molecules that modulate microglial function, further amplifying the inflammatory response (Figure 1). These findings highlight the importance of neuroinflammation in AD pathogenesis. Inflammatory signaling promotes abnormal protein aggregation of Tau and Aβ either by enhancing their aggregation or decreasing their clearance. On the other hand, abnormal protein aggregation dysregulates the brain’s immune cells and impairs their function, thus further contributing to AD pathogenesis.

#### 3.1.3. Synaptic Dysfunction in AD

Synaptic dysfunction has a strong impact on AD. Synapses are specialized junctions between neurons that facilitate the transmission of electrical or chemical signals [65]. The ability of synapses to change their activity where appropriate over time is a fundamental mechanism underlying learning. This phenomenon of synaptic plasticity is profoundly disrupted and affected in different ways in AD [65]. Important mechanisms involved in learning and memory are long-term potentiation (LTP) and long-term depression (LTD) [66]. Those refer to the enhancement of and reduction in synaptic strength and connections. Aβ plaques and oligomers inhibit LTP and enhance LTD [66]. This imbalance leads to synaptic connection weakness and impairs synaptic plasticity and stability, thus affecting cognitive function [66].

Aβ oligomers also damage synapses by binding to N-methyl-D-aspartic acid (NMDA) receptors and causing Ca^2+^ influx and excitotoxicity [67,68]. Additionally, they reduce the expression of essential synaptic proteins, such as synaptophysin and bredrin. Consequently, Aβ oligomers impair neurotransmitter release and synaptic transmission by impairing the key processes of synaptic vesicle cycling [69]. Cholinergic signaling and postsynaptic density protein-95 (PSD-95), which are crucial for synaptic plasticity and cognition, are impaired by the presence of Aβ oligomers [68].

**Figure 2:**
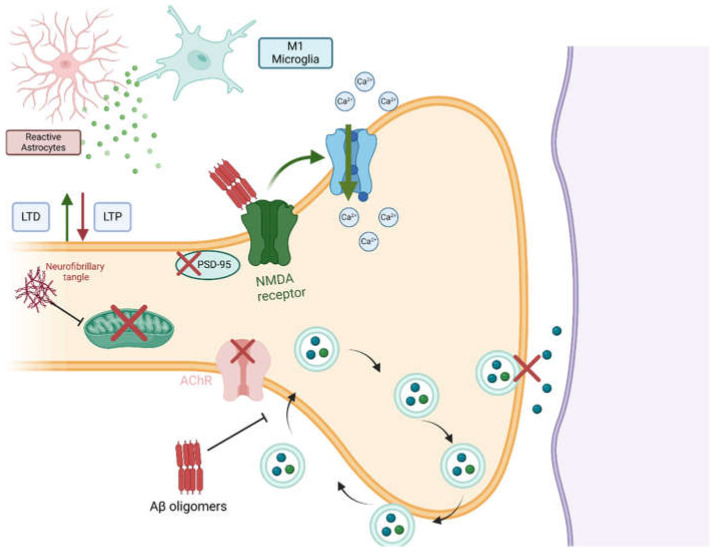
Pathophysiological effects of Aβ and tau on synaptic function in AD. Aβ oligomers disrupt synaptic function through different mechanisms. (1) Aβ oligomers activate NMDA receptors, which, in turn, increase Ca^2+^ influx and induce excitotoxicity. (2) Neurotransmitter release is impaired in AD. This is caused by the interference of Aβ oligomers with synaptic-vesicle recycling and the disruption of cholinergic and PSD-95 signaling, which are required for proper synaptic function. Neurofibrillary tangles affect synaptic function mainly by impairing both mitochondrial and microtubule functions. Lastly, both tau and Aβ aggregates promote neuroinflammation, which activates LTD and inhibits LTP. Created in BioRender. Gharaibeh, A. (2025): https://BioRender.com/m1fy7zf (accessed on 21 May 2025).

Tau pathology/NFTs interfere with axonal transport via their negative effect on microtubules and mitochondrial function, leading to reduced synaptic energy [48]. The combined effect of Aβ and tau in synaptic dysfunction and neuroinflammation results in profound neurodegeneration [70]. Additional effects on synaptic function in AD are exerted by neuroinflammation and the released pro-inflammatory cytokines, which impair synaptic signaling and plasticity [71]. IL-1β has been shown to inhibit LTP and promote LTD, thereby disrupting the balance of synaptic plasticity [71]. Figure 2 summarizes the pathophysiological effects of Aβ and tau on synaptic function in AD.

#### 3.1.4. Peripheral Inflammation and AD

Several studies have mentioned chronic systemic inflammation as a potential risk factor for AD. High levels of inflammatory markers such as C-reactive protein (CRP) increase the risk of developing AD [72,73]. Research reported that AD patients have elevated levels of inflammatory cytokines, including IL-6, and that TNF-α and IL-6 correlate with disease severity [74]. Chronically elevated levels of inflammatory cytokines disrupt the BBB, promoting the entry of immune cells and neurotoxic substances, which contribute to neuroinflammation and AD pathology [74]. Other studies have related peripheral inflammation to the exacerbation of Aβ plaque accumulation. For example, IL-1β and TNF-α increase the production and aggregation of Aβ plaques [50,74]. Peripheral inflammation is also associated with tau hyperphosphorylation, as inflammatory markers also play a role in the activation of kinases [74].

### 3.2. Periodontitis and Alzheimer’s Disease Pathogenesis

Periodontal disease is the inflammation of tissue around the tooth structure [75]. Gingivitis is considered the first stage of periodontal disease and is caused by the accumulation of dental plaque. Inflammation of the gingiva can extend to deeper periodontal structures if left untreated [76]. According to the National Health and Nutrition Examination Survey (NHANES), the prevalence of periodontitis is 42% in dentate adults aged 30 years or older in the United States [12]. Periodontal disease is increasingly recognized as a risk factor for AD. In a case–control study, the researchers found that there is a significant association between periodontal disease and risk of cognitive impairment (OR = 3.04; 95% CI = 1.69 to 5.46) [77]. Others showed a 2.5-fold increased risk of developing dementia in chronic periodontal disease patients in a retrospective matched-cohort study on 2207 patients [78]. This association become more pronounced at 10 years post-chronic periodontal disease diagnosis (adjusted HR, 1.707; *p* = 0.0077) [79].

Furthermore, periodontal disease increased the rate of cognitive decline in patients with AD by six-fold over the course of a 6-month follow-up pilot study with 60 participants [23]. Moreover, tooth loss, commonly caused by periodontitis, was associated with higher prevalence and incidence of dementia in Nun study (OR = 4.3, 95% CI = 1.16 to 15.60, *p* = 0.03) [80]. In a cohort of 468 participants, having more teeth was associated with more favorable AD-related MRI findings, such as infarct, white matter hyperintensity volume, entorhinal cortex volume, and cortical thickness (*p* < 0.01) [81].

The pathogenesis of periodontal disease is a complex, multifaceted process affected by microbial–inflammatory, immunologic, genetic, and environmental factors. Collectively, it is driven by dysregulation between the microbiome and inflammation [10,27,82]. The inflammatory response is not limited to local tissue in periodontal disease but rather spreads and is associated with the systemic inflammatory response and elevated inflammatory markers in the blood [19]. A number of studies have found a link between systemic inflammation and neuroinflammation [83]. The prevailing view of the brain being an immunologically isolated compartment has completely changed [84,85]. Several studies suggested that periodontal disease is a major risk factor for the development of AD [24,79,86]. At the microbiological level, this evidence is supported by the presence of many periodontal pathogens inside the brains of AD patients, such as herpes simplex virus type 1 (HSV-1), actinomyces, oral spirochetes, and, most importantly, *Porphyromonas gingivalis* (*P. gingivalis*) [86,87,88,89].

Moreover, periodontal dysbiosis may alter amyloid pathology in AD. Higher subgingival periodontal dysbiosis is associated with reduced CSF Aβ-42 levels [90]. The spread of periodontal pathogens to the brain is explained via the perivascular spaces, which allow for the dissemination of these pathogens [91,92]. This could be supported by the presence of permeable endothelial junctions in the circumventricular organs (CVOs) [93]. Another hypothesis was proposed when oral treponemas were found in the trigeminal ganglia. This hypothesis claims that olfactory and trigeminal nerves provide potential routes for oral pathogens to enter the brain [94,95,96].

Gingipain, a protease produced by *P. gingivalis*, has the ability to catabolize extracellular matrix components and fibrinogen, in addition to complement proteins and immunoglobulins [97]. It can inhibit the phagocytic ability of macrophages by blocking CD14 receptors [98]. Gingipains also block the CD4 and CD8 receptors of T cells [98]. These effects ease escape from the immune system [98]. Animal models have shown a positive correlation between *P. gingivalis* infection and Aβ 42 accumulation in the brain, and thus, neuroinflammation (*p* < 0.00001) [99,100,101]. Gingipain inhibitors, which decrease the bacterial load in mice, decrease Aβ 1–42 production, further supporting the association between *P. gingivalis* infection and Aβ 42 accumulation [86,102,103]. Additionally, preclinical studies have shown the ability of gingipain inhibitors to prevent the neurotoxic effects of gingipain injected into hippocampal neurons (*p* < 0.05, *n* = 120) [86,101].

Moreover, gingipain levels and antibodies against periodontal pathogens, including *P. gingivalis*, are proportionally related to TNF-α levels and cognitive decline [100,104]. A previous study showed that oral gingipain inhibitors reduced the DNA concentration of *P. gingivalis* in mice with brain *P. gingivalis* infection (90% bacterial load reduction, *p* < 0.0001) [86], as well as reducing Aβ and TNF-α (*p* < 0.01 and *p* < 0.001, respectively) [86]. Additionally, the number of hippocampal neurons increased when gingipain inhibitors were administered [105]. Both *P. gingivalis* and its gingipains could induce tau hyperphosphorylation, and this effect was also linked to *T. denticola* [106,107]. Gingipains activate inflammasomes and microglia, and, when combined with their effect on the complement system, they strongly exacerbate neuroinflammation [108]. Gingipains cleave ApoE into fragments, which are neurotoxic in contrast to the protective role of full-length ApoE [105].

*P. gingivalis* also synthesizes phosphorylated dihydroceramide (PDHC) lipids, such as phosphoethanolamine (PE DHC) and phosphoglycerol dihydroceramide (PG DHC), which influence cellular activity through TLR2 [109]. PG DHC lipids from *P. gingivalis* increase the secretion of soluble Aβ peptides and the expression of APP in specific cells from a mouse model that expresses wild-type human APP in a normal state [109]. It also induces tau hyperphosphorylation in human neuroblastoma cells, contributing to the aging process of these cells by inducing the production of pro-inflammatory cytokines, such as TNF-α and IL-6, and other markers, such as beta-galactosidase and cathepsin B. PG DHC also downregulates a senescence-protective marker called sirtuin-1 [109].

Lipopolysaccharide (LPS) is abundant in the brains of AD patients, and its presence near amyloid plaques and around vessels suggests that it is associated with AβPs (Aβ40/42) [110,111,112]. Specifically, *P. gingivalis* LPS was found in postmortem AD brains but not in healthy individuals [113]. Additionally, when LPS was administered peripherally, both the production of APP and the activity of beta- and gamma-secretases increased, resulting in more Aβ42 in the brain. More interestingly, tau hyperphosphorylation was triggered [114]. Similar findings explained the impact of LPS on the BBB, as it increases Aβ entry and decreases its excretion from the brain [115]. Tau hyperphosphorylation and NFT formation were rapidly exacerbated when LPS was infused in mice with mutated tau [116].

*P. gingivalis* generates two types of lipopolysaccharides, known as O-LPS and A-LPS [117]. A-LPS is particularly noteworthy for its potential involvement in the development of AD [118]. Research showed that neuronal Aβ40/42 levels significantly elevated in mice after an injection of LPS, along with the exacerbation of microglial activation and neuroinflammation [114]. Interestingly, *P. gingivalis* LPS has a higher tendency to bind to astrocytes in the brains of AD patients than in those of non-AD patients [113]. Although less common, the LPS of *Aggregatibacter actinomycetemcomitans* (A. A) also leads to the accumulation of Aβ42 and neuroinflammation, indicating that the effect is not limited to *P. ginivalis* LPS [113,119].

**Figure 3:**
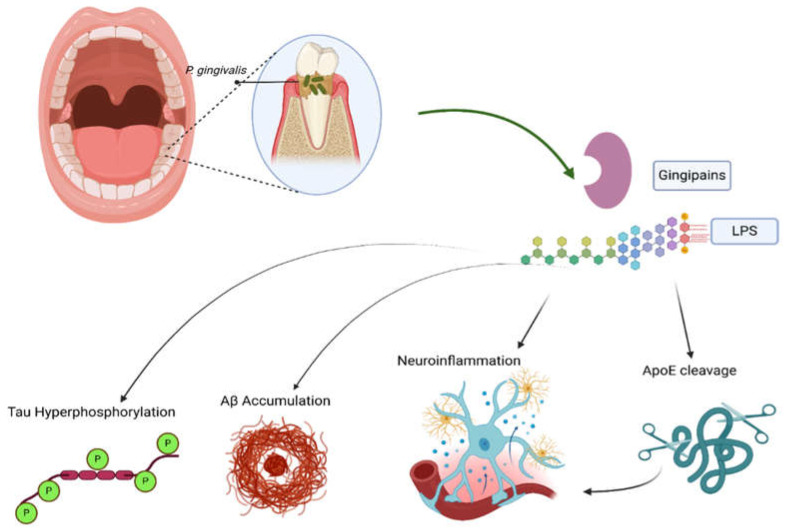
The role of periodontitis in Alzheimer’s disease (AD). *P. gingivalis*, the most common periodontal pathogen, releases gingipains and LPS, both of which promote AD through different mechanisms. These mediators induce tau hyperphosphorylation and Aβ accumulation, in addition to promoting neuroinflammation and ApoE cleavage. The latter further enhances neuroinflammation. Created in BioRender. Gharaibeh, A. (2025): https://BioRender.com/vdmvnqs (accessed on 21 May 2025).

Studies suggest that the neuroinflammation of *P. gingivalis* LPS is primarily dependent on cathepsin B, and this could be targeted to prevent AD cognitive decline in periodontal disease patients [29]. Cathepsin B is a cystine protease that plays a role in the production of IL-1β by activated microglia, in addition to its beta-secretase activity [120,121,122]. Cathepsin B enhances Aβ production, and knocking out cathepsin B blocks the elevation of Aβ1–42 in the brain, highlighting its amyloidogenic effects in the brain. Furthermore, cathepsin B knockout mice exhibited improved memory function and reduced neuroinflammation compared to controls (*p* < 0.07) [102,120,123]. Drawing these threads together, growing evidence supports the role of periodontitis in AD pathology. To summarize, the changes are mainly due to systemic inflammation associated with periodontitis, in addition to some evidence of direct periodontal pathogens’ invasion of the brain. These factors act though manipulating neuroinflammation or direct neurotoxic effects. Key periodontitis-related mediators involved in AD pathogenesis are cathepsin B, gingipain, LPS, TNF, and IL-6. Figure 3 summarizes the main links between the most common periodontal pathogen, namely *P. gingivalis*, and AD.

### 3.3. Inflammatory Biomarkers Linking Periodontitis and Alzheimer’s Disease

Periodontal disease is associated with elevated inflammatory biomarkers, including IL-1, IL-6, TNF-α, and CRP [124,125], which are also elevated in AD [126]. These inflammatory biomarkers can modulate brain function and structure [127]. Furthermore, the correlation between pro-inflammatory mediators and biomarkers of brain Aβ burden underscores the potential role of inflammation in the pathology of AD [128]. Following a periodontal infection, IL-1β and TNF-α are among the first and most important upregulated cytokines. These cytokines induce a pro-inflammatory environment in the brain, contributing to an AD-like pathology and clinical progression of the disease [129]. Researchers conducted a case–control study comparing individuals with no cognitive impairment (n = 131) and patients with mild cognitive impairment (n = 171). In samples from patients with periodontal disease and dementia (70% of whom had AD), the researchers measured a set of periodontal inflammatory biomarkers, including IL-1 receptor antagonist (IL1-Ra), epidermal growth factor (EGF), granulocyte–macrophage colony-stimulating factor (GM-CSF), growth-regulated oncogene (GRO), IL-6, IL-7, IL-8, interferon gamma-induced protein 10 (IP-10), monocyte Chemoattractant Protein-1 (MCP-1), MCP-1α, MCP-1β, and TNF-α. The results showed that all of these biomarkers were lower in the patients with dementia than in the control group, except for IL1-Ra, which was significantly higher in the patients with dementia (*p* < 0.018) [130]. Cathepsin B, as implicated in the previous section, is another serum marker elevated in both periodontal disease and AD, and it is strongly correlated with cognitive decline.

The kynurenine (KYN) pathway, a major pathway of tryptophan (TRP) metabolism in the body, plays a crucial role in neurodegenerative diseases, including AD [131]. KYN can be converted into neuroprotector kynurenic acid (KYNA) or neurotoxic quinolinic acid (QA). An imbalance between KYNA and QA contributes to the progression of neurodegenerative disorders. KYNA protects the brain by blocking excitatory amino acid receptors, specifically NMDA receptors [131]. Interestingly, patients with periodontal disease have significantly higher levels of salivary IL-6, TRP, KYN, KYNA, picolinic acid (PA), and QA than periodontally healthy individuals (n = 40, *p* < 0.05) [132]. Other changes in both salivary and serum KYN/TRP ratios in these patients indicate altered KYN pathway metabolism in periodontal disease [132]. In a small study involving 20 patients with periodontal disease and AD, those who tested positive for *T. denticola* had lower serum neopterin, while those who were positive for T. forsythia had lower serum KYN (positive 1.64 ± 0.17 vs. negative 2.16 ± 0.20; U = 1.980; *p* < 0.05 [133]. This suggests that these periodontal pathogens impact neuroinflammation in AD by modulating adaptive immunity mechanisms [133].

Studies showed that multiple bacterial, viral, and fungal oral pathogens are found in the brains of AD patients [24,86,87,88,89]. Therefore, specific markers of periodontal pathogens could also be used as a screening method for early AD detection [134]. Unfolded p53 (Up53) is produced in non-neuronal cells due to oxidative stress caused by LPS and released into serum. Researchers proposed that the detection of Up53 in serum could be an early diagnostic biomarker for AD, as it appears several years before the clinical symptoms of AD manifest, and it may be linked to bacterial infections, particularly *P. gingivalis* [135]. In a parallel study, Up53 was shown to predict the development of AD within six years in a cohort of 482 patients aged 60–85 years (HR = 2.99 for low versus intermediate/high U-p53AZ variant) [136]. These results indicated a better accuracy than Aβ-PET scan (U-p53AZ achieved area under the curve (AUC) > 98%, and Aβ-PET achieved AUCs 84–93%, *p* < 0.0001 and *p* < 0.001, respectively).

Fungi are also known causes of periodontal disease [137]. The activities of fungal enzymes such as chitinase and stathmin in the CSF have been found to be strong biomarkers for the diagnosis of AD, with accuracy of 85.8%. This percentage is considered higher than clinically standard CSF markers (Aβ = 78.4% and tau = 77.6%), as shown in a comparative study of 94 AD patients, 41 non-AD patients, and 40 controls [137]. These have value in the relationship between periodontal disease and AD, as previous research indicated that chitin-like structures of oral fungi are found in the brains of AD patients [138]. In a study, blood levels of immunoglobulins against periodontal pathogens were measured and found to be higher in AD patients than in healthy patients (72% vs. 38% positive for 1 or more IgG antibody, OR = 6.1) [139]. This is also supported by the correlation between *P. gingivalis* IgG and the degree of cognitive impairment (OR = 2.89 and 95% CI = 1.14 to 7.29 for delayed verbal recall; and OR = 1.95 and 95% CI = 1.22 to 3.11 for impaired subtraction) [140]. Additionally, antibodies against *P. gingivalis*, *T. denticola*, *F. nucleatum*, and *P. intermedia* are statistically significantly elevated in patients with AD [104,141].

Biomarkers that are elevated in both periodontal disease and AD could be monitored as indicators to screen individuals at higher risk of cognitive decline as a result of chronic periodontal disease, thus providing a window into the early detection of AD before cognitive symptoms become apparent [104,142,143,144]. The biomarkers that have been suggested for AD screening in patients with periodontal disease are detailed in Table 1.

Qiu and colleagues found that periodontal disease severity is exacerbated in AD patients, thus suggesting a bidirectional relationship between both diseases [148]. Gingival crevicular fluid (GCF) microbiome showed a relative abundance of 16 species correlated with cognitive function. Additionally, GCF metabolome indicated that some pathogens’ metabolites are significant predictors of AD progression. These include (1) galactinol, (2) sn-glycerol 3 phosphoethanolamine, (3) D-mannitol, (4) 1h-indole-1-pentanoicacid, (5) 3-(1-naphthalenylcarbonyl)-, and (6) L-iditol [148]. Another study compared salivary biomarkers, such as (1) 5-cyclohexadiene-1,2-diol, (2) dodecanoic acid, (3) Cis-3-(1-carboxy-ethyl)-3, (4) and N,N-dimethylethanolamine N-oxide, between AD and non-AD populations with similar periodontal conditions [147]. The results indicated the significant screening value of these biomarkers (n = 60, *p*-values range between <0.001 and 0.05, for all metabolites). AD patients consistently had higher plaque indices (PI) and more bleeding on probing (BOP) on periodontal examinations [147]. In summary, the key mediators, such as inflammatory markers and KYN pathways, not only contribute to AD development but also can be used as potential biomarkers for assessing disease development and progression.

### 3.4. Integrated Early Intervention Strategies for Alzheimer’s Disease and Periodontal Disease

While periodontal disease can often be reversed with timely treatment, AD presents a significant challenge, with considerable medical and financial consequences [149]. Given the previously implicated bidirectional link between these conditions, maintaining oral health may have the potential to protect against cognitive decline by influencing the oral–brain axis [149,150]. This connection could impact disease onset and progression through preventive measures or by targeting specific pathogens [24,86]. Although promising, many therapeutic interventions are still in development, highlighting the importance of early intervention in both periodontal disease and AD to potentially slow progression and improve overall patient well-being [24,151,152]. The intervention strategies are further explained in the sections below.

### 3.5. Bidirectional Pathways in the Prevention of Alzheimer’s Disease and Periodontitis

The rationale for periodontal therapy in AD prevention or treatment is based on the need to control the infectious, immune, inflammatory, and systemic features of periodontal disease, which is rooted in the correlation between chronic periodontal inflammation and neuroinflammation [19,20,23,24,153,154]. By controlling the inflammatory pathways, periodontal therapy may help lower the risk of AD or slow its progression. As periodontal therapy aims to reduce the microbial load and subsequent inflammatory response, it may potentially alleviate the unnecessary burden of AD for certain individuals, hence modifying the host response [17,149,155,156]. A quasi-experimental design study, including 177 periodontally treated patients and 409 untreated ones, indicated that after median observation of 7.3 years, periodontal disease treatment group was associated with less brain atrophy (r = −0.41; 95% CI = −0.70 to −0.12; *p* = 0.0051) [157].

However, cognitive decline might affect the ability of the patient to manage good oral health, which might eventually lead to periodontal disease [158,159]. Oral care strategies include (1) an examination on diagnosis or admission to a nursing home; (2) regular dental screening bi-annually or when needed; (3) and maintaining oral hygiene practices, with an emphasis on the difference in hygiene habits between patients with AD and patients without AD [26,158,159]. Additionally, professional periodontal and adjunctive therapies such as antimicrobial mouthwashes or antibiotics can play an effective role in controlling periodontal infections in AD patients [26,160,161].

#### 3.5.1. Controlling the Bacterial Biofilm

Periodontal disease is primarily initiated by a dysbiotic biofilm composed of Gram-negative anaerobic bacteria and its byproducts, which stimulate an abnormal host immune response and contribute to both local periodontal destruction and systemic inflammation [19,86]. The bacteria responsible for periodontal disease, specifically *P. gingivalis*, contribute to neuroinflammation by promoting the production of pro-inflammatory cytokines, which may cross the BBB and lead to the development of AD [20,103,156,162,163,164]. Hence, the purpose of periodontal therapy approaches is to interrupt the self-sustaining vicious cycle linking microbial dysbiosis with destructive inflammation. This cycle underlies the chronicity of periodontal disease and, in turn, contributes to the progression of AD [27,165]. Controlling the bacterial plaque biofilm could therefore prevent both periodontal disease and cognitive decline [19,23,165].

Regular home care, combined with professional removal of supra- and sub-gingival plaque, is essential for controlling inflammatory periodontal disease [166]. Non-surgical therapy, particularly scaling and root planing (SRP), is the cornerstone of managing periodontal disease [166,167]. This effectively reduces the microbial load and the resulting inflammatory mediators, such as IL-1β and prostaglandin E2 (PGE2) in periodontal tissues, thereby decreasing systemic inflammation and subsequently reducing the risk or severity of AD [27].

Additionally, adjunctive use of dental chemotherapeutic agents such as chlorhexidine gluconate and systemic antibiotics can further enhance the outcomes of SRP, particularly in patients with aggressive forms of periodontal disease [25,26,168,169]. It is also important to assess and treat plaque-retentive factors, such as defective restorations and carious lesions, to prevent disease recurrence and progression [26,170].

#### 3.5.2. Host Modulation Therapy

It is known that periodontal disease is an inflammatory disease associated with systemic inflammation [16,25]. The host immune response is involved in disease development. Host modulation therapy refers to disease treatment where the target of the intervention is the host of the disease, and their function and status are altered rather than the causative agent being directly targeted [27,165]. For this reason, host modulation therapy is another option for treating periodontitis in order to reduce the risk of AD [165]. Such modularity therapy includes non-steroidal anti-inflammatory drugs, anti-cytokine therapy, specialized pro-resolution mediators, probiotics, correcting dysbiosis, complement/homeostatic proteins comprising EGF-like and discoidin-like domains, nuclear metabolic receptor agonists, targeting of adaptive immune cells, approaches for the direct inhibition of periodontal tissue destruction, vaccination, and anti-aging approaches [27].

#### 3.5.3. Assessing the Risk Factors

Highlighting the common risk factors between periodontal disease and AD, such as aging, infection, immunosuppression, smoking [75,171], diabetes [172], genetic predisposition [173], and socioeconomic factors, might pave the way for the development of preventive strategies and treatment approaches. This may potentially modulate the overall risk of AD [156,165,170].

## 4. Conclusions

A plethora of studies have found a link between periodontal disease and AD. These investigations help in better understanding the pathogenesis of AD. Such pioneering thinking sets the stage for new pathway targets and intervention modalities. The importance of understanding this correlation should keep physicians aware of this preventive risk factor, as in practice, it is generally not taken into consideration. The two main ways in which periodontal disease modulates AD are through the induction of systemic inflammation and the direct brain invasion of the oral microbiome. Preventive protocols in dental clinics might be applied depending on the suggested bidirectional link between AD and periodontal disease. Currently, studies suggest a number of potential biomarkers that can be also used to predict or follow AD in periodontitis settings. However, the lack of randomized clinical trials limits causational and prediction conclusions. More effort is needed to further verify these biomarkers in larger studies and clinical trials, and develop an interventional modality to stop the vicious cycle between periodontal disease and AD.

## Figures and Tables

**Table 1:** Biomarkers suggested for AD screening in patients with periodontal disease.

Biomarker(s)	Sample Type	Reference	Year	Study Design	Number of Subjects	Key Finding/s
IL1-Ra	Blood	Gil Montoya, José Antonio et al. [130]	2020	Case–control study	Case (n = 171) and control (n = 131)	Among patients with periodontal disease and dementia, 70% had AD; 29 inflammatory biomarkers were analyzed. IL 1-Ra was significantly elevated in mild periodontitis (*p* < 0.018) and was associated with cognitive impairment (Crude OR = 2.92; 95% CI = 1.14 to 7.46).
Cathepsin B	Blood	Rong, Xianfang et al. [145]	2020	Case–control study	Case (n = 23) and control (n = 45)	Compared with controls, patients with chronic periodontal disease demonstrated elevations in five biomarkers involved in AD pathogenesis, with cathepsin B being the most notable one (42% increase in periodontal disease compared to control, *p* < 0.01; and correlation with MME score, r = −0.874, *p* < 0.001).
Periodontal bacterial antibodies	Blood	Sparks Stein, Pamela et al. [104]	2012	Retrospective study	158	Levels of antibodies against *F. nucleatum* and *P. intermedia* were higher in patients who developed AD (6.1 ± 0.4 in healthy controls vs. 10.0 ± 1.5 in chronic periodontitis; and 6.2 ± 0.4 in healthy controls vs. 15.9 ± 2.2 in chronic periodontitis, respectively. *p* < 0.0001).
Neopterin and KYN	Blood	Leblhuber, Friedrich et al. [132]	2020	Clinical trial	20	Serum levels of neopterin and KYN, which are precursors of adaptive cell metabolites, were low in patients with periodontal disease and AD (positive for periodontal pathogen compared to control negative for periodontal pathogen; positive 6.14 ± 0.65 vs. negative 9.58 ± 0.73 nmol/L; U = 2.533, *p* < 0.01 for neopterin, and positive 1.64 ± 0.17 vs. negative: 2.16 ± 0.20; U = 1.980, *p* < 0.05 for KYN).
Methylmalonic acid (MMA)	Blood	Li, An et al. [146]	2022	Cross-sectional study	1883	Serum MMA was correlated with lower scores of cognitive performance in patients with periodontal disease and cognitive impairment, including AD. (Fully adjusted weighted β coefficient (SE) for CERAD immediate recall −0.229 (0.023) and −0.331 (0.036) for Stage III and Stage IV periodontitis, respectively; *p* < 0.05. For Digit symbol substitution test, fully adjusted weighted β coefficient (SE) −0.139 (0.019) and −0.144 (0.031) for Stage III and Stage IV periodontitis, respectively; *p* < 0.05.)
5-cyclohexadiene-1,2-diol, dodecanoic acid, Cis-3-(1-carboxy-ethyl)-3, and N,N-dimethylethanolamine N-oxide	Salivary	Yang, Yi et al. [147]	2023	Case–control study	Case (n = 30), control (n = 30)	Salivary biomarkers are potential candidates for AD screening in patients with periodontal disease (ROC value = 0.95).
Galactinol, sn-glycerol 3-phosphoethanolamine, D-mannitol, 1 h-indole-1-pentanoic acid, 3-(1-naphthalenylcarbonyl)-, and L-iditol	Salivary	Qiu, Che et al. [148]	2024	Cross-sectional study	64	The levels of these periodontal pathogen’s metabolites in GCF were correlated with AD progression (for galactinol, AUC = 0.98 in AD vs. mild cognitive impairment/normal (aMCI/CN; for sn-glycerol 3-phosphoethanolamine, AUC = 0.98 in AD vs. aMCI and AUC = 0.99 in AD vs. CN).

## Data Availability

Not applicable.

## References

[1] Zhang J., Zhang Y., Wang J., Xia Y., Zhang J., Chen L. (2024). Recent Advances in Alzheimer’s Disease: Mechanisms, Clinical Trials and New Drug Development Strategies. Signal Transduct. Target. Ther..

[2] Knopman D.S., Amieva H., Petersen R.C., Chételat G., Holtzman D.M., Hyman B.T., Nixon R.A., Jones D.T. (2021). Alzheimer Disease. Nat. Rev. Dis. Primers.

[3] Scheltens P., De Strooper B., Kivipelto M., Holstege H., Chételat G., Teunissen C.E., Cummings J., van der Flier W.M. (2021). Alzheimer’s Disease. Lancet.

[4] Dementia. https://www.who.int/news-room/fact-sheets/detail/dementia.

[5] Dementia Statistics|Alzheimer’s Disease International (ADI). https://www.alzint.org/about/dementia-facts-figures/dementia-statistics/.

[6] Wimo A., Seeher K., Cataldi R., Cyhlarova E., Dielemann J.L., Frisell O., Guerchet M., Jönsson L., Malaha A.K., Nichols E. (2023). The worldwide costs of dementia in 2019. Alzheimer’s Dement..

[7] Nichols E., Abd-Allah F., Abdoli A., Abosetugn A.E., Abrha W.A., Abualhasan A., Abu-Gharbieh E., Akinyemi R.O., Alahdab F., GBD 2019 Collaborators (2021). Global mortality from dementia: Application of a new method and results from the Global Burden of Disease Study 2019. Alzheimer’s Dement. Transl. Res. Clin. Interv..

[8] Hardy J.A., Higgins G.A. (1992). Alzheimer’s Disease: The Amyloid Cascade Hypothesis. Science.

[9] Wu D.T., Cho Y.W., Spalti M.D., Bishara M., Nguyen T.T. (2023). The Link between Periodontitis and Alzheimer’s Disease—Emerging Clinical Evidence. Dent. Rev..

[10] Sedghi L.M., Bacino M., Kapila Y.L. (2021). Periodontal Disease: The Good, The Bad, and The Unknown. Front. Cell Infect. Microbiol..

[11] Nazir M.A. (2017). Prevalence of Periodontal Disease, Its Association with Systemic Diseases and Prevention. Int. J. Health Sci..

[12] Eke P.I., Thornton-Evans G.O., Wei L., Borgnakke W.S., Dye B.A., Genco R.J. (2018). Periodontitis in US Adults: National Health and Nutrition Examination Survey 2009–2014. J. Am. Dent. Assoc..

[13] From the Dental Archives: Miller 1891, pt. 1. https://websites.umich.edu/~pfa/denthist/articles/Miller1891.html.

[14] Liccardo D., Marzano F., Carraturo F., Guida M., Femminella G.D., Bencivenga L., Agrimi J., Addonizio A., Melino I., Valletta A. (2020). Potential Bidirectional Relationship Between Periodontitis and Alzheimer’s Disease. Front. Physiol..

[15] Kim J., Amar S. (2006). Periodontal Disease and Systemic Conditions: A Bidirectional Relationship. Odontology.

[16] Arigbede A.O., Babatope B.O., Bamidele M.K. (2012). Periodontitis and Systemic Diseases: A Literature Review. J. Indian Soc. Periodontol..

[17] Bui F.Q., Almeida-da-Silva C.L.C., Huynh B., Trinh A., Liu J., Woodward J., Asadi H., Ojcius D.M. (2019). Association between Periodontal Pathogens and Systemic Disease. Biomed. J..

[18] Hajishengallis G. (2015). Periodontitis: From Microbial Immune Subversion to Systemic Inflammation. Nat. Rev. Immunol..

[19] Hajishengallis G., Chavakis T. (2021). Local and Systemic Mechanisms Linking Periodontal Disease and Inflammatory Comorbidities. Nat. Rev. Immunol..

[20] Kamer A.R., Craig R.G., Dasanayake A.P., Brys M., Glodzik-Sobanska L., de Leon M.J. (2008). Inflammation and Alzheimer’s Disease: Possible Role of Periodontal Diseases. Alzheimer’s Dement..

[21] Cekici A., Kantarci A., Hasturk H., Van Dyke T.E. (2014). Inflammatory and Immune Pathways in the Pathogenesis of Periodontal Disease. Periodontol. 2000.

[22] Offenbacher S., Barros S.P., Beck J.D. (2008). Rethinking Periodontal Inflammation. J. Periodontol..

[23] Ide M., Harris M., Stevens A., Sussams R., Hopkins V., Culliford D., Fuller J., Ibbett P., Raybould R., Thomas R. (2016). Periodontitis and Cognitive Decline in Alzheimer’s Disease. PLoS ONE.

[24] Ryder M.I. (2020). *Porphyromonas gingivalis* and Alzheimer Disease: Recent Findings and Potential Therapies. J. Periodontol..

[25] Gaur S., Agnihotri R. (2015). Alzheimer’s Disease and Chronic Periodontitis: Is There an Association?. Geriatr. Gerontol. Int..

[26] Rolim T.d.S., Fabri G.M.C., Nitrini R., Anghinah R., Teixeira M.J., de Siqueira J.T.T., Cesari J.A.F., de Siqueira S.R.D.T. (2014). Evaluation of Patients with Alzheimer’s Disease before and after Dental Treatment. Arq. Neuropsiquiatr..

[27] Hajishengallis G., Chavakis T., Lambris J.D. (2020). Current Understanding of Periodontal Disease Pathogenesis and Targets for Host-Modulation Therapy. Periodontol. 2000.

[28] Kinane D.F., Stathopoulou P.G., Papapanou P.N. (2017). Periodontal Diseases. Nat. Rev. Dis. Primers.

[29] Wu Z., Ni J., Liu Y., Teeling J.L., Takayama F., Collcutt A., Ibbett P., Nakanishi H. (2017). Cathepsin B Plays a Critical Role in Inducing Alzheimer’s Disease-like Phenotypes Following Chronic Systemic Exposure to Lipopolysaccharide from *Porphyromonas gingivalis* in Mice. Brain Behav. Immun..

[30] Wang R.P.H., Ho Y.S., Leung W.K., Goto T., Chang R.C.C. (2019). Systemic Inflammation Linking Chronic Periodontitis to Cognitive Decline. Brain Behav. Immun..

[31] Sehar U., Rawat P., Reddy A.P., Kopel J., Reddy P.H. (2022). Amyloid Beta in Aging and Alzheimer’s Disease. Int. J. Mol. Sci..

[32] Selkoe D.J., Hardy J. (2016). The Amyloid Hypothesis of Alzheimer’s Disease at 25 Years. EMBO Mol. Med..

[33] Vassar R., Bennett B.D., Babu-Khan S., Kahn S., Mendiaz E.A., Denis P., Teplow D.B., Ross S., Amarante P., Loeloff R. (1999). Beta-Secretase Cleavage of Alzheimer’s Amyloid Precursor Protein by the Transmembrane Aspartic Protease BACE. Science.

[34] Haass C., Selkoe D.J. (2007). Soluble Protein Oligomers in Neurodegeneration: Lessons from the Alzheimer’s Amyloid Beta-Peptide. Nat. Rev. Mol. Cell Biol..

[35] Jarrett J.T., Berger E.P., Lansbury P.T. (1993). The Carboxy Terminus of the Beta Amyloid Protein Is Critical for the Seeding of Amyloid Formation: Implications for the Pathogenesis of Alzheimer’s Disease. Biochemistry.

[36] Finder V.H., Glockshuber R. (2007). Amyloid-Beta Aggregation. Neurodegener. Dis..

[37] Lambert M.P., Barlow A.K., Chromy B.A., Edwards C., Freed R., Liosatos M., Morgan T.E., Rozovsky I., Trommer B., Viola K.L. (1998). Diffusible, Nonfibrillar Ligands Derived from Abeta1-42 Are Potent Central Nervous System Neurotoxins. Proc. Natl. Acad. Sci. USA.

[38] Weingarten M.D., Lockwood A.H., Hwo S.Y., Kirschner M.W. (1975). A Protein Factor Essential for Microtubule Assembly. Proc. Natl. Acad. Sci. USA.

[39] Wang J.Z., Liu F. (2008). Microtubule-Associated Protein Tau in Development, Degeneration and Protection of Neurons. Prog. Neurobiol..

[40] Buée L., Bussière T., Buée-Scherrer V., Delacourte A., Hof P.R. (2000). Tau Protein Isoforms, Phosphorylation and Role in Neurodegenerative Disorders. Brain Res. Rev..

[41] Ashton N.J., Brum W.S., Molfetta G.D., Benedet A.L., Arslan B., Jonaitis E., Langhough R.E., Cody K., Wilson R., Carlsson C.M. (2024). Diagnostic Accuracy of a Plasma Phosphorylated Tau 217 Immunoassay for Alzheimer Disease Pathology. JAMA Neurol..

[42] Therriault J., Janelidze S., Benedet A.L., Ashton N.J., Arranz Martínez J., Gonzalez-Escalante A., Bellaver B., Alcolea D., Vrillon A., Karim H. (2024). Diagnosis of Alzheimer’s Disease Using Plasma Biomarkers Adjusted to Clinical Probability. Nat. Aging.

[43] Iqbal K., Liu F., Gong C.-X., Grundke-Iqbal I. (2010). Tau in Alzheimer Disease and Related Tauopathies. Curr. Alzheimer Res..

[44] Goedert M., Spillantini M.G., Potier M.C., Ulrich J., Crowther R.A. (1989). Cloning and Sequencing of the CDNA Encoding an Isoform of Microtubule-Associated Protein Tau Containing Four Tandem Repeats: Differential Expression of Tau Protein MRNAs in Human Brain. EMBO J..

[45] Grundke-Iqbal I., Iqbal K., Tung Y.C., Quinlan M., Wisniewski H.M., Binder L.I. (1986). Abnormal Phosphorylation of the Microtubule-Associated Protein Tau (Tau) in Alzheimer Cytoskeletal Pathology. Proc. Natl. Acad. Sci. USA.

[46] Lee V.M.Y., Goedert M., Trojanowski J.Q. (2001). Neurodegenerative Tauopathies. Annu. Rev. Neurosci..

[47] Braak H., Braak E. (1991). Neuropathological Stageing of Alzheimer-Related Changes. Acta Neuropathol..

[48] Ittner L.M., Götz J. (2011). Amyloid-β and Tau--a Toxic Pas de Deux in Alzheimer’s Disease. Nat. Rev. Neurosci..

[49] Pooler A.M., Polydoro M., Maury E.A., Nicholls S.B., Reddy S.M., Wegmann S., William C., Saqran L., Cagsal-Getkin O., Pitstick R. (2015). Amyloid Accelerates Tau Propagation and Toxicity in a Model of Early Alzheimer’s Disease. Acta Neuropathol. Commun..

[50] Heneka M.T., Golenbock D.T., Latz E. (2015). Innate Immunity in Alzheimer’s Disease. Nat. Immunol..

[51] Prinz M., Tay T.L., Wolf Y., Jung S. (2014). Microglia: Unique and Common Features with Other Tissue Macrophages. Acta Neuropathol..

[52] De Strooper B., Karran E. (2016). The Cellular Phase of Alzheimer’s Disease. Cell.

[53] Hickman S.E., Kingery N.D., Ohsumi T.K., Borowsky M.L., Wang L.C., Means T.K., El Khoury J. (2013). The Microglial Sensome Revealed by Direct RNA Sequencing. Nat. Neurosci..

[54] Tang Y., Le W. (2016). Differential Roles of M1 and M2 Microglia in Neurodegenerative Diseases. Mol. Neurobiol..

[55] Morales I., Jiménez J.M., Mancilla M., Maccioni R.B. (2013). Tau Oligomers and Fibrils Induce Activation of Microglial Cells. J. Alzheimer’s Dis..

[56] Asai H., Ikezu S., Tsunoda S., Medalla M., Luebke J., Haydar T., Wolozin B., Butovsky O., Kügler S., Ikezu T. (2015). Depletion of Microglia and Inhibition of Exosome Synthesis Halt Tau Propagation. Nat. Neurosci..

[57] Frost B., Diamond M.I. (2010). Prion-like Mechanisms in Neurodegenerative Diseases. Nat. Rev. Neurosci..

[58] Sofroniew M.V., Vinters H.V. (2010). Astrocytes: Biology and Pathology. Acta Neuropathol..

[59] Maragakis N.J., Rothstein J.D. (2006). Mechanisms of Disease: Astrocytes in Neurodegenerative Disease. Nat. Clin. Pract. Neurol..

[60] Verkhratsky A. (2006). Glial Calcium Signaling in Physiology and Pathophysiology. Acta Pharmacol. Sin..

[61] Hopp S.C., Lin Y., Oakley D., Roe A.D., Devos S.L., Hanlon D., Hyman B.T. (2018). The Role of Microglia in Processing and Spreading of Bioactive Tau Seeds in Alzheimer’s Disease. J. Neuroinflamm..

[62] Puangmalai N., Bhatt N., Montalbano M., Sengupta U., Gaikwad S., Ventura F., McAllen S., Ellsworth A., Garcia S., Kayed R. (2020). Internalization Mechanisms of Brain-Derived Tau Oligomers from Patients with Alzheimer’s Disease, Progressive Supranuclear Palsy and Dementia with Lewy Bodies. Cell Death Dis..

[63] Sidoryk-Wegrzynowicz M., Wegrzynowicz M., Lee E., Bowman A.B., Aschner M. (2011). Role of Astrocytes in Brain Function and Disease. Toxicol. Pathol..

[64] Heneka M.T., Kummer M.P., Stutz A., Delekate A., Schwartz S., Vieira-Saecker A., Griep A., Axt D., Remus A., Tzeng T.C. (2013). NLRP3 Is Activated in Alzheimer’s Disease and Contributes to Pathology in APP/PS1 Mice. Nature.

[65] Selkoe D.J. (2002). Alzheimer’s Disease Is a Synaptic Failure. Science.

[66] Shankar G.M., Li S., Mehta T.H., Garcia-Munoz A., Shepardson N.E., Smith I., Brett F.M., Farrell M.A., Rowan M.J., Lemere C.A. (2008). Amyloid-Beta Protein Dimers Isolated Directly from Alzheimer’s Brains Impair Synaptic Plasticity and Memory. Nat. Med..

[67] De Felice F.G., Velasco P.T., Lambert M.P., Viola K., Fernandez S.J., Ferreira S.T., Klein W.L. (2007). Abeta Oligomers Induce Neuronal Oxidative Stress through an N-Methyl-D-Aspartate Receptor-Dependent Mechanism That Is Blocked by the Alzheimer Drug Memantine. J. Biol. Chem..

[68] Snyder E.M., Nong Y., Almeida C.G., Paul S., Moran T., Choi E.Y., Nairn A.C., Salter M.W., Lombroso P.J., Gouras G.K. (2005). Regulation of NMDA Receptor Trafficking by Amyloid-Beta. Nat. Neurosci..

[69] Wang Z., Jackson R.J., Hong W., Taylor W.M., Corbett G.T., Moreno A., Liu W., Li S., Frosch M.P., Slutsky I. (2017). Human Brain-Derived Aβ Oligomers Bind to Synapses and Disrupt Synaptic Activity in a Manner That Requires APP. J. Neurosci..

[70] Bloom G.S. (2014). Amyloid-β and Tau: The Trigger and Bullet in Alzheimer Disease Pathogenesis. JAMA Neurol..

[71] Ross F.M., Allan S.M., Rothwell N.J., Verkhratsky A. (2003). A Dual Role for Interleukin-1 in LTP in Mouse Hippocampal Slices. J. Neuroimmunol..

[72] Khatri A., Prakash O., Agarwal R., Kushwaha S. (2024). Systemic Inflammatory Markers and Their Association with Alzheimer’s Disease: A Cross-Sectional Analysis. Indian. J. Psychiatry.

[73] Kravitz B.A., Corrada M.M., Kawas C.H. (2009). Elevated C-Reactive Protein Levels Are Associated with Prevalent Dementia in the Oldest-Old. Alzheimer’s Dement..

[74] Lyra e Silva N.M., Gonçalves R.A., Pascoal T.A., Lima-Filho R.A.S., Resende E. (2021). de P. F.; Vieira, E.L.M.; Teixeira, A.L.; de Souza, L.C.; Peny, J.A.; Fortuna, J.T.S.; et al. Pro-Inflammatory Interleukin-6 Signaling Links Cognitive Impairments and Peripheral Metabolic Alterations in Alzheimer’s Disease. Transl. Psychiatry.

[75] Meyle J., Chapple I. (2015). Molecular Aspects of the Pathogenesis of Periodontitis. Periodontol. 2000.

[76] Gingivitis-StatPearls-NCBI Bookshelf. https://www.ncbi.nlm.nih.gov/books/NBK557422/.

[77] Gil-Montoya J.A., Sanchez-Lara I., Carnero-Pardo C., Fornieles F., Montes J., Vilchez R., Burgos J.S., Gonzalez-Moles M.A., Barrios R., Bravo M. (2015). Is Periodontitis a Risk Factor for Cognitive Impairment and Dementia? A Case-Control Study. J. Periodontol..

[78] Tzeng N.S., Chung C.H., Yeh C.B., Huang R.Y., Yuh D.Y., Huang S.Y., Lu R.B., Chang H.A., Kao Y.C., Chiang W.S. (2016). Are Chronic Periodontitis and Gingivitis Associated with Dementia? A Nationwide, Retrospective, Matched-Cohort Study in Taiwan. Neuroepidemiology.

[79] Chen C.K., Wu Y.T., Chang Y.C. (2017). Association between Chronic Periodontitis and the R isk of Alzheimer’s Disease: A Retrospective, Population-Based, Matched-Cohort Study. Alzheimer’s Res. Ther..

[80] Stein P.S., Desrosiers M., Donegan S.J., Yepes J.F., Kryscio R.J. (2007). Tooth Loss, Dementia and Neuropathology in the Nun Study. J. Am. Dent. Assoc..

[81] Rubinstein T., Brickman A.M., Cheng B., Burkett S., Park H., Annavajhala M.K., Uhlemann A.C., Andrews H., Gutierrez J., Paster B.J. (2024). Periodontitis and Brain Magnetic Resonance Imaging Markers of Alzheimer’s Disease and Cognitive Aging. Alzheimer’s Dement..

[82] Avula H., Chakravarthy Y. (2022). Models of Periodontal Disease Pathogenesis: A Journey through Time. J. Indian Soc. Periodontol..

[83] Perry V.H., Cunningham C., Holmes C. (2007). Systemic Infections and Inflammation Affect Chronic Neurodegeneration. Nat. Rev. Immunol..

[84] Holmes C. (2013). Review: Systemic Inflammation and Alzheimer’s Disease. Neuropathol. Appl. Neurobiol..

[85] Holmes C., Cunningham C., Zotova E., Woolford J., Dean C., Kerr S., Culliford D., Perry V.H. (2009). Systemic Inflammation and Disease Progression in Alzheimer Disease. Neurology.

[86] Dominy S.S., Lynch C., Ermini F., Benedyk M., Marczyk A., Konradi A., Nguyen M., Haditsch U., Raha D., Griffin C. (2019). *Porphyromonas gingivalis* in Alzheimer’s Disease Brains: Evidence for Disease Causation and Treatment with Small-Molecule Inhibitors. Sci. Adv..

[87] Itzhaki R.F., Wozniak M.A. (2006). Herpes Simplex Virus Type 1, Apolipoprotein E, and Cholesterol: A Dangerous Liaison in Alzheimer’s Disease and Other Disorders. Prog. Lipid Res..

[88] Siddiqui H., Eribe E.R., Singhrao S.K., Olsen I. (2019). High Throughput Sequencing Detect Gingivitis And Periodontal Oral Bacteria In Alzheimer’s Disease Autopsy Brains. Neuro Res..

[89] Emery D.C., Shoemark D.K., Batstone T.E., Waterfall C.M., Coghill J.A., Cerajewska T.L., Davies M., West N.X., Allen S.J. (2017). 16S RRNA Next Generation Sequencing Analysis Shows Bacteria in Alzheimer’s Post-Mortem Brain. Front. Aging Neurosci..

[90] Kamer A.R., Pushalkar S., Gulivindala D., Butler T., Li Y., Annam K.R.C., Glodzik L., Ballman K.V., Corby P.M., Blennow K. (2021). Periodontal dysbiosis associates with reduced CSF Aβ42 in cognitively normal elderly. Alzheimer’s Dement..

[91] Stamatovic S., Keep R., Andjelkovic A. (2008). Brain Endothelial Cell-Cell Junctions: How to “Open” the Blood Brain Barrier. Curr. Neuropharmacol..

[92] Wardlaw J.M., Benveniste H., Nedergaard M., Zlokovic B.V., Mestre H., Lee H., Doubal F.N., Brown R., Ramirez J., MacIntosh B.J. (2020). Perivascular Spaces in the Brain: Anatomy, Physiology and Pathology. Nat. Rev. Neurol..

[93] Ganong W.F. (2000). Circumventricular Organs: Definition and Role in the Regulation of Endocrine and Autonomic Function. Clin. Exp. Pharmacol. Physiol..

[94] Danielyan L., Schäfer R., von Ameln-Mayerhofer A., Buadze M., Geisler J., Klopfer T., Burkhardt U., Proksch B., Verleysdonk S., Ayturan M. (2009). Intranasal Delivery of Cells to the Brain. Eur. J. Cell Biol..

[95] Johnson N.J., Hanson L.R., Frey W.H. (2010). Trigeminal Pathways Deliver a Low Molecular Weight Drug from the Nose to the Brain and Orofacial Structures. Mol. Pharm..

[96] Riviere G., Riviere K.H., Smith K.S. (2002). Molecular and Immunological Evidence of Oral Treponema in the Human Brain and Their Association with Alzheimer’s Disease. Oral. Microbiol. Immunol..

[97] Singhrao S.K., Harding A., Poole S., Kesavalu L., Crean S.J. (2015). *Porphyromonas gingivalis* Periodontal Infection and Its Putative Links with Alzheimer’s Disease. Mediat. Inflamm..

[98] Zheng S., Yu S., Fan X., Zhang Y., Sun Y., Lin L., Wang H., Pan Y., Li C. (2021). *Porphyromonas gingivalis* Survival Skills: Immune Evasion. J. Periodontal Res..

[99] Díaz-Zúñiga J., More J., Melgar-Rodríguez S., Jiménez-Unión M., Villalobos-Orchard F., Muñoz-Manríquez C., Monasterio G., Valdés J.L., Vernal R., Paula-Lima A. (2020). Alzheimer’s Disease-Like Pathology Triggered by *Porphyromonas gingivalis* in Wild Type Rats Is Serotype Dependent. Front. Immunol..

[100] Ilievski V., Zuchowska P.K., Green S.J., Toth P.T., Ragozzino M.E., Le K., Aljewari H.W., O’Brien-Simpson N.M., Reynolds E.C., Watanabe K. (2018). Chronic Oral Application of a Periodontal Pathogen Results in Brain Inflammation, Neurodegeneration and Amyloid Beta Production in Wild Type Mice. PLoS ONE.

[101] Poole S., Singhrao S.K., Chukkapalli S., Rivera M., Velsko I., Kesavalu L., Crean S. (2015). Active Invasion of *Porphyromonas gingivalis* and Infection-Induced Complement Activation in ApoE-/- Mice Brains. J. Alzheimer’s Dis..

[102] Nakanishi H., Nonaka S., Wu Z. (2020). Microglial Cathepsin B and *Porphyromonas gingivalis* Gingipains as Potential Therapeutic Targets for Sporadic Alzheimer’s Disease. CNS Neurol. Disord. Drug Targets.

[103] Haditsch U., Roth T., Rodriguez L., Hancock S., Cecere T., Nguyen M., Arastu-Kapur S., Broce S., Raha D., Lynch C.C. (2020). Alzheimer’s Disease-Like Neurodegeneration in *Porphyromonas gingivalis* Infected Neurons with Persistent Expression of Active Gingipains. J. Alzheimer’s Dis..

[104] Sparks Stein P., Steffen M.J., Smith C., Jicha G., Ebersole J.L., Abner E., Dawson D. (2012). Serum Antibodies to Periodontal Pathogens Are a Risk Factor for Alzheimer’s Disease. Alzheimer’s Dement..

[105] Sabbagh M.N., Decourt B. (2022). COR388 (Atuzaginstat): An Investigational Gingipain Inhibitor for the Treatment of Alzheimer Disease. Expert. Opin. Investig. Drugs.

[106] Tang Z., Cheng X., Su X., Wu L., Cai Q., Wu H. (2022). Treponema Denticola Induces Alzheimer-Like Tau Hyperphosphorylation by Activating Hippocampal Neuroinflammation in Mice. J. Dent. Res..

[107] Kanagasingam S., Chukkapalli S.S., Welbury R., Singhrao S.K. (2020). *Porphyromonas gingivalis* Is a Strong Risk Factor for Alzheimer’s Disease. J. Alzheimer’s Dis. Rep..

[108] Olsen I. (2021). *Porphyromonas gingivalis*-Induced Neuroinflammation in Alzheimer’s Disease. Front. Neurosci..

[109] Yamada C., Akkaoui J., Ho A., Duarte C., Deth R., Kawai T., Nichols F., Lakshmana M.K., Movila A. (2020). Potential Role of Phosphoglycerol Dihydroceramide Produced by Periodontal Pathogen *Porphyromonas gingivalis* in the Pathogenesis of Alzheimer’s Disease. Front. Immunol..

[110] Choe K., Park J.S., Park H.Y., Tahir M., Park T.J., Kim M.O. (2024). Lupeol Protect against LPS-Induced Neuroinflammation and Amyloid Beta in Adult Mouse Hippocampus. Front. Nutr..

[111] Godbout J.P., Chen J., Abraham J., Richwine A.F., Berg B.M., Kelley K.W., Johnson R.W. (2005). Exaggerated Neuroinflammation and Sickness Behavior in Aged Mice Following Activation of the Peripheral Innate Immune System. FASEB J..

[112] Zhan X., Stamova B., Sharp F.R. (2018). Lipopolysaccharide Associates with Amyloid Plaques, Neurons and Oligodendrocytes in Alzheimer’s Disease Brain: A Review. Front. Aging Neurosci..

[113] Poole S., Singhrao S.K., Kesavalu L., Curtis M.A., Crean S.J. (2013). Determining the Presence of Periodontopathic Virulence Factors in Short-Term Postmortem Alzheimer’s Disease Brain Tissue. J. Alzheimer’s Dis..

[114] Lee J.W., Lee Y.K., Yuk D.Y., Choi D.Y., Ban S.B., Oh K.W., Hong J.T. (2008). Neuro-Inflammation Induced by Lipopolysaccharide Causes Cognitive Impairment through Enhancement of Beta-Amyloid Generation. J. Neuroinflamm..

[115] Jaeger L.B., Dohgu S., Sultana R., Lynch J.L., Owen J.B., Erickson M.A., Shah G.N., Price T.O., Fleegal-Demotta M.A., Butterfiled D.A. (2009). Lipopolysaccharide Alters the Blood-Brain Barrier Transport of Amyloid Beta Protein: A Mechanism for Inflammation in the Progression of Alzheimer’s Disease. Brain Behav. Immun..

[116] Lee D.C., Rizer J., Selenica M.L.B., Reid P., Kraft C., Johnson A., Blair L., Gordon M.N., Dickey C.A., Morgan D. (2010). LPS- Induced Inflammation Exacerbates Phospho-Tau Pathology in RTg4510 Mice. J. Neuroinflamm..

[117] Rangarajan M., Aduse-Opoku J., Paramonov N.A., Hashim A., Curtis M.A. (2017). Hemin Binding by *Porphyromonas gingivalis* Strains Is Dependent on the Presence of A-LPS. Mol. Oral. Microbiol..

[118] Olsen I., Singhrao S.K. (2018). Importance of Heterogeneity in Porhyromonas Gingivalis Lipopolysaccharide Lipid A in Tissue Specific Inflammatory Signalling. J. Oral. Microbiol..

[119] Díaz-Zúñiga J., Muñoz Y., Melgar-Rodríguez S., More J., Bruna B., Lobos P., Monasterio G., Vernal R., Paula-Lima A. (2019). Serotype b of Aggregatibacter Actinomycetemcomitans Triggers Pro-Inflammatory Responses and Amyloid Beta Secretion in Hippocampal Cells: A Novel Link between Periodontitis and Alzheimer’s Disease?. J. Oral. Microbiol..

[120] Hook V.Y.H., Kindy M., Reinheckel T., Peters C., Hook G. (2009). Genetic Cathepsin B Deficiency Reduces β-Amyloid in Transgenic Mice Expressing Human Wild-Type Amyloid Precursor Protein. Biochem. Biophys. Res. Commun..

[121] Halle A., Hornung V., Petzold G.C., Stewart C.R., Monks B.G., Reinheckel T., Fitzgerald K.A., Latz E., Moore K.J., Golenbock D.T. (2008). The NALP3 Inflammasome Is Involved in the Innate Immune Response to Amyloid-Beta. Nat. Immunol..

[122] Wu Z., Sun L., Hashioka S., Yu S., Schwab C., Okada R., Hayashi Y., McGeer P.L., Nakanishi H. (2013). Differential Pathways for Interleukin-1β Production Activated by Chromogranin A and Amyloid β in Microglia. Neurobiol. Aging.

[123] Hook G., Reinheckel T., Ni J., Wu Z., Kindy M., Peters C., Hook V. (2022). Cathepsin B Gene Knockout Improves Behavioral Deficits and Reduces Pathology in Models of Neurologic Disorders. Pharmacol. Rev..

[124] Kornman K.S., Page R.C., Tonetti M.S. (1997). The Host Response to the Microbial Challenge in Periodontitis: Assembling the Players. Periodontol. 2000.

[125] Gomes-Filho I.S., Freitas Coelho J.M., da Cruz S.S., Passos J.S., Teixeira de Freitas C.O., Aragão Farias N.S., Amorim da Silva R., Silva Pereira M.N., Lima T.L., Barreto M.L. (2011). Chronic Periodontitis and C-Reactive Protein Levels. J. Periodontol..

[126] Koyama A., O’Brien J., Weuve J., Blacker D., Metti A.L., Yaffe K. (2013). The Role of Peripheral Inflammatory Markers in Dementia and Alzheimer’s Disease: A Meta-Analysis. J. Gerontol. A Biol. Sci. Med. Sci..

[127] Warren K.N., Beason-Held L.L., Carlson O., Egan J.M., An Y., Doshi J., Davatzikos C., Ferrucci L., Resnick S.M. (2018). Elevated Markers of Inflammation Are Associated With Longitudinal Changes in Brain Function in Older Adults. J. Gerontol. A Biol. Sci. Med. Sci..

[128] Kiddle S.J., Thambisetty M., Simmons A., Riddoch-Contreras J., Hye A., Westman E., Pike I., Ward M., Johnston C., Lupton M.K. (2012). Plasma Based Markers of [11C] PiB-PET Brain Amyloid Burden. PLoS ONE.

[129] Wang R.P.H., Huang J., Chan K.W.Y., Leung W.K., Goto T., Ho Y.S., Chang R.C.C. (2023). IL-1β and TNF-α Play an Important Role in Modulating the Risk of Periodontitis and Alzheimer’s Disease. J. Neuroinflamm..

[130] Gil Montoya J.A., Barrios R., Sanchez-Lara I., Ramos P., Carnero C., Fornieles F., Montes J., Santana S., Luna J.d.D., Gonzalez-Moles M.A. (2020). Systemic Inflammatory Impact of Periodontitis on Cognitive Impairment. Gerodontology.

[131] Németh H., Toldi J., Vécsei L. (2006). Kynurenines, Parkinson’s Disease and Other Neurodegenerative Disorders: Preclinical and Clinical Studies. J. Neural Transm. Suppl..

[132] Kurgan Ş., Önder C., Balcı N., Akdoğan N., Altıngöz S.M., Serdar M.A., Günhan M. (2022). Influence of Periodontal Inflammation on Tryptophan-Kynurenine Metabolism: A Cross-Sectional Study. Clin. Oral. Investig..

[133] Leblhuber F., Huemer J., Steiner K., Gostner J.M., Fuchs D. (2020). Knock-on Effect of Periodontitis to the Pathogenesis of Alzheimer’s Disease?. Wien. Klin. Wochenschr..

[134] Olsen I., Singhrao S.K. (2015). Can Oral Infection Be a Risk Factor for Alzheimer’s Disease?. J. Oral. Microbiol..

[135] French P.W. (2022). Unfolded P53 in Non-Neuronal Cells Supports Bacterial Etiology of Alzheimer’s Disease. Neural Regen. Res..

[136] Piccirella S., Van Neste L., Fowler C., Masters C.L., Fripp J., Doecke J.D., Xiong C., Uberti D., Kinnon P. (2022). A Conformational Variant of P53 (U-P53AZ) as Blood-Based Biomarker for the Prediction of the Onset of Symptomatic Alzheimer’s Disease. J. Prev. Alzheimer’s Dis..

[137] Watabe-Rudolph M., Song Z., Lausser L., Schnack C., Begus-Nahrmann Y., Scheithauer M.O., Rettinger G., Otto M., Tumani H., Thal D.R. (2012). Chitinase Enzyme Activity in CSF Is a Powerful Biomarker of Alzheimer Disease. Neurology.

[138] Castellani R.J., Perry G., Smith M.A. (2007). The Role of Novel Chitin-like Polysaccharides in Alzheimer Disease. Neurotox. Res..

[139] Kamer A.R., Craig R.G., Pirraglia E., Dasanayake A.P., Norman R.G., Boylan R.J., Nehorayoff A., Glodzik L., Brys M., de Leon M.J. (2009). TNF-Alpha and Antibodies to Periodontal Bacteria Discriminate between Alzheimer’s Disease Patients and Normal Subjects. J. Neuroimmunol..

[140] Noble J.M., Borrell L.N., Papapanou P.N., Elkind M.S.V., Scarmeas N., Wright C.B. (2009). Periodontitis Is Associated with Cognitive Impairment among Older Adults: Analysis of NHANES-III. J. Neurol. Neurosurg. Psychiatry.

[141] Al-Sharqi A.J., Abdulkareem A. (2024). Microbiological and Salivary Biomarkers Successfully Predict Site-Specific and Whole-Mouth Outcomes of Nonsurgical Periodontal Treatment. J. Clin. Med..

[142] Loos B.G. (2005). Systemic Markers of Inflammation in Periodontitis. J. Periodontol..

[143] Papapanou P.N. (2015). Systemic Effects of Periodontitis: Lessons Learned from Research on Atherosclerotic Vascular Disease and Adverse Pregnancy Outcomes. Int. Dent. J..

[144] Thakkar A., Vora A., Kaur G., Akhtar J. (2023). Dysbiosis and Alzheimer’s Disease: Role of Probiotics, Prebiotics and Synbiotics. Naunyn Schmiedebergs Arch. Pharmacol..

[145] Rong X., Xiang L., Li Y., Yang H., Chen W., Li L., Liang D., Zhou X. (2020). Chronic Periodontitis and Alzheimer Disease: A Putative Link of Serum Proteins Identification by 2D-DIGE Proteomics. Front. Aging Neurosci..

[146] Li A., Du M., Chen Y., Marks L.A.M., Visser A., Xu S., Tjakkes G.H.E. (2022). Periodontitis and Cognitive Impairment in Older Adults: The Mediating Role of Mitochondrial Dysfunction. J. Periodontol..

[147] Yang Y., Lv J., Bai H., Ren L., Yang J., Ding Y., Liu C., Chen X. (2023). Periodontal Status and Saliva Metabolic Signature in Patients with Alzheimer’s Disease. J. Alzheimer’s Dis..

[148] Qiu C., Zhou W., Shen H., Wang J., Tang R., Wang T., Xie X., Hong B., Ren R., Wang G. (2024). Profiles of Subgingival Microbiomes and Gingival Crevicular Metabolic Signatures in Patients with Amnestic Mild Cognitive Impairment and Alzheimer’s Disease. Alzheimer’s Res. Ther..

[149] Li R., Wang J., Xiong W., Luo Y., Feng H., Zhou H., Peng Y., He Y., Ye Q. (2024). The Oral-Brain Axis: Can Periodontal Pathogens Trigger the Onset and Progression of Alzheimer’s Disease?. Front. Microbiol..

[150] Lu J., Zhang S., Huang Y., Qian J., Tan B., Qian X., Zhuang J., Zou X., Li Y., Yan F. (2022). Periodontitis-Related Salivary Microbiota Aggravates Alzheimer’s Disease via Gut-Brain Axis Crosstalk. Gut Microbes.

[151] Sait A.M., Day P.J.R. (2024). Interconnections between the Gut Microbiome and Alzheimer’s Disease: Mechanisms and Therapeutic Potential. Int. J. Mol. Sci..

[152] Ma Y.Y., Li X., Yu J.T., Wang Y.J. (2024). Therapeutics for Neurodegenerative Diseases by Targeting the Gut Microbiome: From Bench to Bedside. Transl. Neurodegener..

[153] Martínez-García M., Hernández-Lemus E. (2021). Periodontal Inflammation and Systemic Diseases: An Overview. Front. Physiol..

[154] Guo H., Chang S., Pi X., Hua F., Jiang H., Liu C., Du M. (2021). The Effect of Periodontitis on Dementia and Cognitive Impairment: A Meta-Analysis. Int. J. Environ. Res. Public Health.

[155] Chen H.-L., Wu D.-R., Lien S., Lin C.-H. (2023). Association between periodontitis treatment and dementia in Taiwanese adults. Open Access BMC Oral Health.

[156] Harding A., Gonder U., Robinson S.J., Crean S.J., Singhrao S.K. (2017). Exploring the Association between Alzheimer’s Disease, Oral Health, Microbial Endocrinology and Nutrition. Front. Aging Neurosci..

[157] Schwahn C., Frenzel S., Holtfreter B., Van der Auwera S., Pink C., Bülow R., Friedrich N., Völzke H., Biffar R., Kocher T. (2022). Effect of Periodontal Treatment on Preclinical Alzheimer’s Disease—Results of a Trial Emulation Approach. Alzheimer’s Dement..

[158] Farsai P.S. (2021). Cognitive Impairment in Older Adults and Oral Health Considerations: Treatment and Management. Dent. Clin. N. Am..

[159] Brennan L.J., Strauss J. (2014). Cognitive Impairment in Older Adults and Oral Health Considerations: Treatment and Management. Dent. Clin. N. Am..

[160] Chalmers J., Pearson A. (2005). Oral hygiene care for residents with dementia: A literature review. J. Adv. Nurs..

[161] Rozas N.S., Sadowsky J.M., Jeter C.B. (2017). Strategies to Improve Dental Health in Elderly Patients with Cognitive Impairment: A Systematic Review. J. Am. Dent. Assoc..

[162] Singhrao S.K., Olsen I. (2019). Assessing the Role of *Porphyromonas gingivalis* in Periodontitis to Determine a Causative Relationship with Alzheimer’s Disease. J. Oral. Microbiol..

[163] Kamer A.R., Fortea J.O., Videla S., Mayoral A., Janal M., Carmona-Iragui M., Benejam B., Craig R.G., Saxena D., Corby P. (2016). Periodontal Disease’s Contribution to Alzheimer’s Disease Progression in Down Syndrome. Alzheimer’s Dement. Diagn. Assess. Dis. Monit..

[164] Harding A., Robinson S., Crean S., Singhrao S.K. (2017). Can Better Management of Periodontal Disease Delay the Onset and Progression of Alzheimer’s Disease?. J. Alzheimer’s Dis..

[165] Harding A., Kanagasingam S., Welbury R., Singhrao S.K. (2022). Periodontitis as a Risk Factor for Alzheimer’s Disease: The Experimental Journey So Far, with Hope of Therapy. Adv. Exp. Med. Biol..

[166] Cobb C.M. (2002). Clinical Significance of Non-Surgical Periodontal Therapy: An Evidence-Based Perspective of Scaling and Root Planing. J. Clin. Periodontol..

[167] Haffajee A.D., Cugini M.A., Dibart S., Smith C., Kent R.L., Socransky S.S. (1997). The Effect of SRP on the Clinical and Microbiological Parameters of Periodontal Diseases. J. Clin. Periodontol..

[168] Drisko C.H. (2001). Nonsurgical Periodontal Therapy. Periodontol. 2000.

[169] Zhang M., Mi N., Ying Z., Lin X., Jin Y. (2023). Advances in the Prevention and Treatment of Alzheimer’s Disease Based on Oral Bacteria. Front. Psychiatry.

[170] Kwon T.H., Lamster I.B., Levin L. (2021). Current Concepts in the Management of Periodontitis. Int. Dent. J..

[171] Peters R., Poulter R., Warner J., Beckett N., Burch L., Bulpitt C. (2008). Smoking, Dementia and Cognitive Decline in the Elderly, a Systematic Review. BMC Geriatr..

[172] Shaik M., Ahmad S., Gan S., Abuzenadah A., Ahmad E., Tabrez S., Ahmed F., Kamal M. (2014). How Do Periodontal Infections Affect the Onset and Progression of Alzheimer’s Disease?. CNS Neurol. Disord. Drug Targets.

[173] Okamoto N., Morikawa M., Amano N., Yanagi M., Takasawa S., Kurumatani N. (2017). Effects of Tooth Loss and the Apolipoprotein E ε4 Allele on Mild Memory Impairment in the Fujiwara-Kyo Study of Japan: A Nested Case-Control Study. J. Alzheimer’s Dis..

